# Differences that matter: developing critical insights into discourses of patient-centeredness

**DOI:** 10.1007/s11019-016-9712-7

**Published:** 2016-06-01

**Authors:** Bettine Pluut

**Affiliations:** Utrecht University School of Governance, Bijlhouwerstraat 6, 3511 ZC Utrecht, Netherlands

**Keywords:** Patient-centered care, Patient empowerment, Shared decision-making, Discourse Analysis, Health Information Exchange

## Abstract

Patient-centeredness can be considered a popular, and at the same time “fuzzy”, concept. Scientists have proposed different definitions and models. The present article studies scientific publications that discuss the meaning of patient-centeredness to identify different “discourses” of patient-centeredness. Three discourses are presented; the first is labelled as “caring for patients”, the second as “empowering patients” and the third as “being responsive”. Each of these discourses has different things to say about (a) the why of patient-centeredness; (b) the patient’s identity; (c) the role of the healthcare professional; (d) responsibilities for medical decision-making, and (e) the role of health information. This article compares and contrasts the discourses in ways that allow us to see differences that matter for practitioners in healthcare. On the basis of a relational constructionist philosophy, it is argued that discursive diversity is both an inevitable and a potentially valuable aspect of conversations in healthcare. We are therefore invited to center the challenge of dealing with diversity in productive ways. This article ends with a discussion of the practical implications of the discourse analysis for projects that aim to make healthcare more patient-centered. Debates on patient-centered “Health Information Exchange” are used to explain the need for a recognition of different discourses of patient-centeredness and a reflexive stance towards them.

## Introduction

In the past few decades, a universal plea for patient-centeredness has been heard in medicine (Bensing 2000). It has become a central value in healthcare that is believed to increase the social, psychological, cultural and ethical sensitivities of human encounters (Hughes et al. 2008). For many healthcare practitioners, acting in a way that furthers the interests of patients and their families has thus become a guiding principle to address their ethical sense of the ‘good’ (McGrath et al. 2006; Duggan et al. 2006).

Despite - or maybe due to - the increased attention for patient-centeredness, it can be considered a “fuzzy concept” (Bensing 2000; Illingworth 2010). Scientists have complained about the lack of a globally accepted definition or model because they feel it hinders research into the outcomes and effectiveness of patient-centered care practices (Hobbs 2009; Mead and Bower 2000; Robinson et al. 2008). Hence, it is not surprising that many researchers have performed a review of the literature on patient-centeredness and proposed one model or definition (e.g. Epstein et al. 2005; Jayadevappa and Chhatre 2011; Lusk and Fater 2013). In this article, however, I refrain from making (strong) claims about what patient-centeredness should mean and instead promote the view that much is to be gained from identifying and working with different orientations towards patient-centered care practices.

In the present article, 34 scientific journal articles on patient-centered medical encounters are studied through a discourse analytic approach to identify different orientations towards patient-centeredness, i.e. different “discourses”. The process of studying discourses, Discourse Analysis, challenges taken-for-granted understandings and helps us see “differences that matter” (Phillips and Hardy 2002; Deetz 1996). In this article, I make discourses of patient-centeredness the subject of critical reflection. Further, I argue that the diversity in discourses of patient-centeredness is a healthy and inescapable aspect of conversations in healthcare. Consequently, this article discusses the challenge of dealing with dissensus on how patients could and should be centered. This pluralist orientation is substantiated through a reflexive analysis of projects aimed at improving the exchange of health information, as I believe these serve as insightful illustrations of contexts that would benefit from a recognition of different discourses of patient-centeredness, as well as a reflexive stance towards them.

## A discourse analytic approach

There is a large body of literature on discourse theory and discourse analysis, embracing different meta-theoretical positions (Phillips and Hardy 2002). Relational constructionism is the “meta-theory” or “philosophy” that underlies the Discourse Analysis that is presented in this article. It can be seen as a particular version of social constructionism (McNamee and Hosking 2012; Gergen 1999). One of the characteristics of relational constructionism is that it centres *relational processes* and focuses on the social production of *multiple local realities* (Hosking 2011). Rather then referring to a geographical location, these local realities need to be understood as standing in contrasting relation to the more traditional focus on a universal, stable reality ‘out there’ (Hosking 2007). Practitioners from different countries who enact the same discourse of patient-centeredness could, for instance, be seen as a local community with its local-historical and local-cultural constructions of “good healthcare”, on the basis of local criteria of ethics and quality (McNamee and Hosking 2012). Discourse can be seen as a tool that helps us study local realties. In this article, discourse is defined as *an interrelated set of texts and practices that bring an object into being* (based on Phillips and Hardy 2002, p. 3, quoting Parker). Discourses can be seen as local constructions of people, things, or concepts. Relational constructionism invites a critical orientation towards discourses and assumes that discourses are political: they involve a social positioning of actors and legitimize certain relational practices, whilst discrediting others (Hardy and Phillips 1999). In the present article, discourses of patient-centeredness are studied for how they position doctors and patients and how they construct the power relations between them.

## Selected articles and method of analysis

The discourse analysis (henceforth “DA”) that is presented in this article is based on a sample of 34 articles, published in English language scientific journals between 2004 and 2014. The appendix lists the selected articles, mainly published in the domain of patient education, doctor-patient communication, nursing, healthcare quality, the sociology of illness, and healthcare ethics. All articles explicitly discuss the (‘right’) meaning of patient-centeredness and/or promote a (‘best’) definition or model of patient-centeredness. For practical reasons, the selection of articles was restricted to discussions of patient-centeredness in relation to the medical encounter.

To find scientific writings in medically oriented journals, the widely used Pubmed database was searched for articles that were published between 2004 and 2014. Articles were searched for having ‘patient-centered*’, patient-centred*’, ‘patient empowerment’ or ‘shared decision-making’ in the title or abstract. These terms seem to be most often used in texts that address the meaning of patient-centeredness, and they seem to be most used as search terms in literature reviews on the subject of patient-centered care as well. Articles were included in the analysis if (a) they were in English; (b) there was a digital version available; (c) the title or abstract suggested that the article contained explicit reflections on the meaning of patient-centeredness; and (d) the article discussed patient-centeredness in relation to encounters between healthcare professionals and patients. Through snowballing, additional articles that helped to identify differences amongst discourses were identified.

DA usually involves an “iterative” and “emergent” analytical process, which was also the case for the DA that is presented in this article (Wetherell et al. 2001). Given the interest in different constructions of patient-centeredness, the focus was on themes that were discussed differently by different texts (i.e. fragments, sections, whole articles). This resulted in the emergence of three discourses that construct an interrelated set of themes differently. At some point, “saturation” seemed to occur; newly added articles did not suggest new themes or discourses. The names of the discourses are inspired by the language used in the texts that construct a particular discourse.

## Overview of the three discourses

Table 1 overviews the most important characteristics of the three discourses of patient-centeredness as they emerged out of the analysis of scientific articles.

**Table 1:** Overview of three discourses of patient-centeredness on the basis of five themes

	Discourse 1: caring for patients	Discourse 2: empowering patients	Discourse 3: being responsive
Why be patient-centered?	- Alleviate vulnerabilities - Lessen suffering - Improve quality of diagnosis	- Facilitate self-management - Respect patient autonomy	- Depends on the person and/or context - Center processes of communication
Patient identity: key words	- In need of care - Vulnerable - Experiencing individual	- Right to control - Capable of decision-making - Autonomous	- Multiple identities - Varying preferences
What is the primary role of the healthcare professional?	- To care	- To facilitate, advise, and coach patients in decision-making - To activate the will and ability to make decisions	- To be responsive to (and thus accept) the patient’s needs, values and preferences with respect to the content of encounters, the style of communication, and involvement in decision-making
Who is primarily responsible for decision-making?	- The healthcare professional	- The patient, who can decide to share responsibilities	- The healthcare professional and/or patient
What is the role of health information?	- For a good diagnosis - For compliance	- For a good diagnosis - For choice	- Tailored information - For compliance, for choice or withhold information

As the table shows, the themes of the discourses each draw our attention to important differences in how texts construct patient-centeredness.

The first theme, the why of patient-centeredness, invites us to study how patient-centeredness is advocated on different grounds. Self (such as a particular discourse of patient-centeredness) is often constructed by creating a devalued Other - a realm that is not us, not what we believe, not true, or not good (Gergen et al. 2001). This theme thus draws our attention to how a particular discourse is promoted on the basis of a process of “self-other differentiation” (McNamee and Hosking 2012). Consequently, the importance of caring for patients as “whole persons” (“Self” in discourse one) is promoted by pointing at the downsides of disease-centeredness (a “significant Other” for discourse one), and how disease-centeredness overlooks the psychosocial aspects of illness. The practice of empowering patients (“Self” in discourse two) is promoted by underlining the importance of patient autonomy and self-management, and by critiquing paternalistic approaches to medical encounters (a significant Other for discourse two). Last, the importance of “being responsive” (“Self” in discourse three) is constructed by critiquing texts that neglect the particularities of each medical encounter, such as the discourses of “caring for patients” and “empowering patients” (significant others for discourse three).

The second theme, constructions of patient identity, builds on the assumption of identity as a local and ambiguous construction that has important political implications (Phillips and Hardy 2002). For each discourse, patient identities are constructed differently: the first assumes a vulnerable patient that is in need of help, the second constructs patients as autonomous beings, and texts that we can see as indicative of the third discourse emphasize a diversity in patient identities. Each of these identity constructions has different implications for our thinking about patient-centered medical encounters, in particular the responsibilities for medical decision-making.

The third theme helps us reflect on the ways in which the ‘right’ role of the healthcare professional is constructed. As will become clear, different constructions of the patient’s identity give way to different notions of the ideal role of the healthcare provider. When the patient is mainly constructed as vulnerable and in need of care, this gives way to discussions of the healthcare professional as a caring individual (discourse one). When patients are seen as autonomous individuals that are responsible for ‘self-management’, the healthcare professional’s task is reframed as an empowering coach, facilitator or advisor (discourse two). When multiple patient identities are assumed, the healthcare professional faces the challenge of being responsive to the patient’s preferences with respect to the content of encounters, the style of communication, and involvement in decision-making (discourse three).

The fourth theme, responsibility for decision-making, helps us see how the different discourses have something different to say about who needs to be responsible for determining the ‘right’ diagnosis, and for making decisions about treatments. This is rather a controversial and dominant topic in texts that discuss patient-centeredness in relation to medical encounters. Some emphasize the responsibilities of healthcare professionals in their task of caring (typical for discourse one), others center the patient’s responsibility for making decisions about his/her treatment (indicative of the second discourse), and still others argue that this mainly depends on the (interplay between) the encounter and the persons that participate in them (illustrative for discourse three).

The fifth and last theme is that of Health Information, which here refers to (written and spoken) information about the patient, the care process, the patient’s illness and (possible) treatments. The increasing availability of health information for patients has fuelled discussions on the role of patients in healthcare (Henwood et al. 2003). When patient-centeredness is constructed as a process of “caring for patients”, the focus is on eliciting the information that is needed for a good diagnosis and for compliance to the healthcare professional’s advice. When patient-centeredness is spoken of as a process of “empowering patients”, the focus in on how the patient can be informed in ways that help him/her make (shared) decisions. If patient-centeredness is constructed as a challenge of “being responsive”, the focus is on how to determine the right role of health information and on tailoring information for patients.

In the following paragraphs the three discourses are presented. Each description starts with a short summary and is then followed by an integrated discussion of the five themes for each discourse.

## Discourse 1: caring for patients

### Discourse summary

In this first discourse, patient-centeredness is constructed as a process during which the healthcare professional cares for the patient as a whole person. This need for holistic caring emerged out of a critique of disease-centeredness and is rooted in a construction of the patient as an experiencing individual that is vulnerable and in need of help. The main reason for being patient-centered is to alleviate vulnerabilities, to lessen suffering, and to increase the quality of care through a diagnosis that takes the whole person into account. The primary role of health information is discussed in relation to compliance to the treatment that is suggested by the healthcare professional. In addition, it is considered vital for the effectiveness of medical encounters that patients provide healthcare professionals with information about their illness experience and social context.

### Constructing patient-centeredness as “caring for patients”

In this discourse most attention goes out to the ways in which healthcare professionals can truly care for patients. Patients are assumed to have a “compromised physiological state” and a “threatened identity” because they often experience a lack of control and/or feel alienated (Hobbs 2009, p. 55). For that reason, healthcare professionals need to reduce suffering and alleviate vulnerabilities through a process of caring. Hudon et al. (2012) have, for instance, stressed the importance of legitimizing the patient’s experience of chronic illness, which means healthcare professionals invite patients to express their doubts, concerns, and feelings of loss and grief. When the struggles with their illness are acknowledged, patients are assumed to feel “relieved” (Hudon et al. 2012, p. 173).

In the texts that construct patient-centeredness as a process of caring, healthcare professionals are asked to adopt the biopsychosocial model, which Tanenbaum (2013) summarizes as caring for whole persons instead of their parts. The healthcare professional needs to see patients as persons who experience their illness individually, within their unique social setting (Illingworth 2010). In other words, healthcare professionals are urged to take a holistic perspective and to pay attention to the patient’s illness experience:

Holistic care […] recognizes and values whole persons as well as the interdependence of their parts […]. The whole person is described as the biological, social, psychological, and spiritual aspects of a person. Providing holistic care allows the clinician to better understand how an illness affects the entire person. […] (Morgan and Yoder 2012, p. 8).

According to Hudon et al. (2012), understanding the whole person can include knowing about the patients’ life context (e.g. family, work, religion, culture, social support) as well as personal development stages (life history and personal and developmental issues). In this context, Illingworth explains how models can be used to elicit the patient’s perspective. By using the “FIFE-model”, for instance, healthcare professionals can gain an understanding of the patient’s Feelings about their problems, Ideas about what is wrong, Effect of the illness on functionality, and Expectations of the doctor (Illingworth 2010, quoting Stewart et al. 1995).

Abley (2012) links the importance of alleviating vulnerabilities to the healthcare professional’s task of understanding the individual patient and states that healthcare professionals must be careful not to make assumptions about when patients feel vulnerable. Older people’s views of their own vulnerability, for example, may be different to the views of professionals. True caring for patients thus requires that healthcare professionals try to understand each patient’s vulnerabilities, so they can adequately intervene to reduce vulnerability.

Texts that I have identified as indicative of a caring discourse emphasize the importance of therapeutic engagement between healthcare professional and patient.

Therapeutic engagement is a cyclical process based on the development of trust. […] When the process of therapeutic engagement is successful, the patient receives […] care that lessens suffering and ensures that their needs are met (Hobbs 2009, p. 57–58)

As this quote illustrates, patient-centeredness as a process of “caring for patients” often seems to be promoted on humanistic grounds: lessening the patient’s suffering, and meeting his/her needs seem to be constructed as ‘good things’ in their own right (Hobbs 2009; Hudon et al. 2012). In addition, holistic caring is linked to the effectiveness of care. Texts that construct patient-centeredness as caring refer to the work of Engel (1977), Mishler (1984), and Stewart et al. (1995), who have explored how an understanding of the person’s illness experience and social context leads to a better diagnosis and better decisions about treatments (Illingworth 2010; Hudon et al. 2012). Patient-centeredness as “caring for patients” is thus often promoted by critiquing the biomedical model: it is argued that the exploratory perspective on illness needs to be broadened to include psychosocial aspects (Bensing 2000; Mead and Bower 2000).

Texts that construct this discourse seem to assume that the needs of patients exceed their capacity for self-care. Patients are talked about as passive participants who are “vulnerable” and “suffering” (Hobbs 2009, p. 55). You could say that the patient’s main task is to share information about him- or herself, so that diagnoses and treatment decisions are based on both medical and psychosocial data (Mishler 1984; Engel 1977). Patients are seen as the “recipients of medical decisions and prescriptions”, which Aujoulat et al. (2007) describe as a “compliance-oriented approach”. Consequently, healthcare professionals need to provide patients with the information that enables them to comply with the healthcare professional’s decisions (Henwood et al. 2003). This passive identity construction of patients is criticized and reconstructed in the second discourse.

## Discourse 2: empowering patients

### Discourse summary

In this second discourse, labelled as “empowering patients”, patient-centeredness is constructed as a process that needs to empower patients to be involved in their own care and to manage their own health. Patient-centeredness as empowerment is promoted on both moral grounds (because it recognizes autonomy and self-control) and functional grounds (it is assumed to have a health-promoting effect). Patients can be considered empowered when they make autonomous decisions, as well as when they (decide to) share responsibilities for decision-making with their healthcare professional. The primary role of the healthcare professional is to coach, facilitate and advice patients in their roles as self-managers and (co) decision-makers. Providing patients with access to relevant health information is often spoken of as a vital aspect of the process of empowerment (“information for choice”).

### Constructing patient-centeredness as “empowering patients”

Whereas the texts defining the first discourse focus on the vulnerabilities and needs of patients and the doctor’s agency, other texts centre the rights, abilities and responsibilities of patients (Aujoulat et al. 2007). Here we find frequent use of words such as ‘engagement in care’, ‘self-management’, ‘self-control’ or ‘shared control’, ‘being informed’, ‘autonomy’, ‘patient participation’, ‘shared decision-making’, ‘informed decision-making’ and ‘empowerment’ (Illingworth 2010; Makoul and Clayman 2006; Moumjid et al. 2007; Sacristán 2013). For this reason, I am calling this discourse “empowering patients”.

This discourse is based on the fundamental idea that patients should gain and take responsibility over their life, health, and care. Patients are assumed to have an “innate ability” to master their health (Anderson and Funnell 2010, p. 279). Moreover, it is assumed that most patients not only can, but also want to take responsibility for their health:

Experience has shown that when patients know they have options for the best treatment, screening test, or diagnostic procedure, most of them will want to participate with their clinicians in making the choice. This interest is shared by patients worldwide (Barry and Edgman-Levitan 2012, p. 9).

The empowerment discourse of patient-centeredness can be seen as a move away from doctor-centeredness and focuses on a redistribution of control and responsibilities from doctor to patient (De Haes 2006; Bensing 2000). Patient-centeredness as empowerment is promoted on moral grounds because it recognizes the importance of self-determination and patient autonomy (Taylor 2009):

The underlying philosophy of an empowerment-based approach […] views human beings as having the right and ability to choose by and for themselves. Self-determination therefore appears to be a strong guiding principle of empowerment-based interventions. […] The key features of an empowerment-approach are ideology-driven and concern choice and responsibility on the one hand, and skills development so as to become more competent in […] dealing with one’s disease, life and environment on the other hand (Aujoulat et al. 2007, p. 15 and p. 17).

In addition, texts that construct patient-centeredness as “empowering patients” also link empowerment to the effectiveness of care, because it can increase the quality of decisions and disease management:

Patients provide 98 % of their own diabetes care. […] Because patients are in control of their daily self-management decisions, they are responsible for those decisions and the resulting consequences (Anderson and Funnell 2010, p. 278).

Empowerment as patient-centered care is spoken of as particularly relevant in the case of chronic illness (mainly because of the importance of medication management) (Pulvirenti et al. 2014; Anderson and Funnell 2010), and in those cases in which there is no ‘clear-cut’ answer to a medical question (Anderson and Funnell 2010; Aujoulat et al. 2007; De Haes 2006; Sacristán 2013; Légaré and Witteman 2013):

The most important attribute of patient-centered care is the active engagement of patients when fateful health care decisions must be made. […] For most medical decisions […] more than one reasonable path forward exists […], and different paths entail different combinations of possible therapeutic effects and side effects. […] In such cases, patient involvement in decision-making adds substantial value (Barry and Edgman-Levitan 2012, p. 780).

Despite the shared focus on patient involvement in decision-making, the advocates of an empowerment approach vary in the amount of responsibility they assign to patients. See, for instance, how Moumjid et al. distinguish informed decision-making from shared decision-making:

Informed decision-making is a process that implies that the physician’s knowledge is transferred to the patient, who then has the knowledge and preferences necessary to make a decision. The patient is thus the sole decision maker, whereas in SDM (shared decision-making), the physician and the patient mutually inform each other to reach a common agreement on the decision to implement (Moumjid et al. 2007, p. 541).

This quote shows how some promoters of the empowerment discourse are more radical than others: some believe that it is the patient who should eventually make decisions and others argue that the doctor and empowered patient should make decisions together. Furthermore, various scientists emphasize that patients should still be considered empowered if they choose freely to delegate responsibility for decision-making to their doctor. According to them, empowerment is about patients being self-conscious and their behaviour being self-directed (Anderson and Funnell 2010). In other words, the key to a process of empowerment is that it is guided by the principle of self-determination (Aujoulat et al. 2007).

Even though texts on empowerment de-centre the healthcare professional’s responsibility and emphasize the importance of self-management beyond the medical encounter, healthcare professionals are still believed to have an important task in the process of empowerment (Légaré and Witteman 2013; Pulvirenti et al. 2014). Healthcare professionals need to act as the patient’s advisor, coach, or facilitator and they need to activate the patient’s desire and ability to be engaged (Sacristán 2013).

The role of the HCP [healthcare professional] is to serve as a facilitator and expert resource […] HCPs are responsible for helping patients achieve their goals and overcome barriers through education, appropriate care recommendations, expert advice, self-reflection, and social and self-management support” (Anderson and Funnell 2010, p. 280).

Health information has a central place in this second discourse. The general line of argument is that patients need to be informed in order to be able to make decisions with respect to their health situation, an idea that has been captured by the widely adopted notion of ‘informed choice’ (Henwood et al. 2003). Piper and De Haes have summarized the link between empowerment and information provision as follows:

Information giving then […] is not empowering per se, or in any way an empowerment endpoint […], but it is an important starting point and patients cannot be empowered without information (Piper 2010, p. 176).

One cannot be involved in decision making without being well informed (De Haes 2006, p. 293).

In the empowerment discourse ‘information for choice’ - rather than ‘information for compliance’ - is considered an important patient right (Henwood et al. 2003; Pulvirenti et al. 2014). It is argued that, if patients lack “health literacy skills”, then this ability to “critically analyse information” needs to be built by healthcare professionals and/or the broader healthcare system (Pulvirenti et al. 2014, p. 308–309).

The information that patients need in order to make decisions, such as information on “conditions/treatment options/treatment plans” (Davis et al. 2005, p. 954), can come from a wide range of sources, varying from books, the Internet and information leaflets provided by healthcare professionals (Berwick 2009). In addition, patients can be facilitated in their role as (shared) decision maker through access to their medical record. In his self-called “extreme view on patient-centered care”, Berwick even argues that the access rights to medical records should be reversed: medical records should belong to patients, and “clinicians rather than patients would need to have permission to gain access to them” (Berwick 2009, p. 561).

Légaré and Witteman put high trust in decision support technologies, because they can facilitate the process of shared decision-making, and because they can help to solve health literacy issues:

[S]hared decision making assumes that both the provider and the patient require access to […] the best available medical evidence relevant to the decision […] Accordingly, shared decision making often involves the use of patient decision aids—structured tools such as booklets or interactive online applications that summarize the available evidence relevant to a given decision and that, ideally, help the patient clarify her or his relevant values. […] Well-crafted tools for shared decision making can […] be specifically designed to meet the needs of people with lower health literacy […] (Légaré and Witteman 2013, p. 277 and p. 280).

Sacristán (2013), on the other hand, argues that even with decision aids, empowerment places new demands on patients. They need to take on greater responsibilities and develop a greater level of literacy so that they are able to benefit from these aids.

## Discourse 3: being responsive

### Discourse summary

In this discourse, patient-centeredness is constructed as a process of responsive communication. Texts that construct patient-centeredness as “being responsive” are characterized by a plea for context-dependency, which is rooted in the assumption of multiple patient identities and a focus on the particularities of every specific encounter. It is argued that not all patients prefer to be cared for on the basis of a biospychosocial model and/or do not want to be empowered. In other words, patient-centeredness as “being responsive” is often promoted by critiquing the first two discourses. If it comes down to providing patients with health information, health care professionals need to take the context and patient’s values, need and preferences into account to determine what information is provided, for what reason, and how.

### Constructing patient-centeredness as “being responsive”

Context-dependency is key to this discourse, which implies that those who promote it give up the idea that there is one best way to carry out medical encounters:

Rather than […] a specific set of behaviours, responsiveness and informed flexibility should be considered fundamental qualities of PCC [patient-centered care, added by author](Epstein et al. 2005, p. 1518).

Responsiveness is discussed in relation to the content of the medical encounter (e.g. whether or not psychosocial issues are addressed), to the patient’s preferences with respect to communication styles (e.g. a friendly or more to-the-point communication style), and as a form of sensitivity to the patient’s wishes with respect to involvement in decision-making (how much responsibility s/he wishes).

The first, responsiveness with respect to the content of the medical encounter, implies that if a patient prefers the biomedical model over the biopsychosocial model, a medical encounter can still be considered patient-centered:

Patient-centered medicine alludes to a humanistic, biopsychosocial perspective as opposed to the conventional perspective of the biomedical model. This need not necessarily be the case though. At any one time communication may shift from one domain of this model to another and still be construed as patient-centered as long as the transition is negotiated, i.e. both doctor and patient agree to behave in this way (Taylor 2009, p 151).

The second argument for responsiveness is based on the belief that different patients prefer different styles of communication. Bergman and Connaughton (2013) argue, for example, that the healthcare professional’s sensitivity to communication preferences can be critical to whether patients perceive an encounter as patient-centered. In a similar vein, De Haes (2006) suggests that the effectiveness of the physicians’ communication may depend on the person in front of them.

The third, being responsive to the patient’s preferences for decision-making, is explained by Fineberg in the following quote:

Just as it would be sub-optimal to impose a paternalistic style on […] patients who prefer to be involved in their care decisions, it is equally sub-optimal to force a shared role on the [patients] who prefer either to make autonomous decisions or to have their doctor decide what would be best. The […] goal should be to prepare physicians to identify and assess each patient’s preferences and to adopt the style that meets the needs of each individual patient (Fineberg 2012, p. 2).

Likewise, Jayadevappa and Chhatre (2011) argue that patient-centered care is about tailored care, which means that physicians and nurses accept that some patients prefer to actively participate in decision making, whilst others opt for a more passive role and wish to defer decisions to their physicians.

Some authors emphasize differences in patient characteristics when promoting the idea of responsiveness, whilst others point at contextual factors. As an illustration of the former, Bergman and Connaughton (2013) have related the importance of responsiveness to the challenge of cultural diversity. On the basis of a study of the preferences of Hispanic prenatal care patients, they argue that patient-centeredness requires cultural sensitivity:

Sometimes referred to as cross-cultural differences, divisions in sociocultural patterns often produce dilemmas between medical experts and patients […]. These challenges may relate to differences in communication styles or spiritual beliefs, as well as the role that hierarchy and respect play in active participation in doctor– patient interactions […] Each patient and provider comes to the health care encounter with his/her own beliefs and expectations guided by a particular culture of health. […] A worthwhile goal would be to avoid defining and implementing patient centered communications as a one-size-fits-all approach (Bergman and Connaughton 2013, p. 791 and 797)

In a similar vein, Jayadevappa and Chhatre (2011) argue that cultural competence needs to be factored into patient-centered care. Among other things, understanding the meaning of culture can help healthcare professionals to understand and be responsive to patient beliefs, preferences and needs, and consequently, to build rapport and trust. De Haes (2006) points at anxiety traits as an explanation for different patient preferences with respect to communication styles, and relatedly, for differences in the effectiveness of these styles. Last, Lawrence and Kinn (2012) argue that a definition of patient-centeredness needs to be condition-specific, as patients with different diseases might value different interventions and outcomes.

Another person-based argument for responsiveness is that some patients can be considered experts in their illness, whilst others lack the medical knowledge and/or skills that are necessary for medical decision-making (Fineberg 2012; Jayadevappa and Chhatre 2011; Taylor 2009; Badcott 2005; Agarwal and Murinson 2012).

Authors that emphasize contextual factors when promoting the idea of responsive communication argue that the same patient does not always prefer the same amount of responsibility in medical decision-making. In other words, a patient’s preferences with respect to involvement in medical decision-making may vary for different contexts or disease/life phases (De Haes 2006; Holmström and Röing 2010; Sinding et al. 2010):

It is neither an ethical lapse nor a character flaw for a patient to prefer to have the doctor decide what to do. Nor is it irrational for a patient to want to be in control of decisions, with the physician or physicians acting as advisors and guides. Indeed, the same patient at different stages of life or facing different circumstances of illness may prefer at times to be a passive recipient of care, an active partner in decision-making, or fully in control of the choices to be made. […] the capable and humane clinician will adapt a role and mode of interaction that suits the needs of each patient at each particular time. This is patient-centered decision making. It requires a readiness on the part of physicians to involve the patient in shared decision-making without feeling uniformly compelled to do so (Fineberg 2012, p. 2).

As becomes clear from the above arguments, in this discourse healthcare professionals are asked to adapt their style of communication to the person and context and negotiate responsibilities accordingly (Illingworth 2010; Taylor 2009). Ishikawa, Hashimoto and Kuchu suggest that patients need to do the same. They argue that it is important to focus on the relational process between healthcare professional and patient and how both share their perspectives, so that they can come to construct new ideas, mutual agreement and shared decisions - to which they also refer as a “shared mind” (Ishikawa et al. 2013, p. 150).

The plea for responsiveness also manifests itself in the ways in which the advocates of this discourse speak of health information. Being patient-centered also means centering patients’ differing information needs:

The behaviors that go along with patient-centered care are […] also having the intuition or judgment necessary to tailor explorative interventions, giving or withholding information and sharing power to the needs of an individual patient (De Haes 2006, p. 276).

Intervention can be a sensitive and supportive process that genuinely seeks to respond to patient preferences in informing (Piper 2010, p. 176).

Responsive information provision means that if a healthcare professional chooses to withhold information on the basis of an understanding of the patient’s cognitive capacity, health literacy, values, and desire for information, such a judgment can still be seen as “patient-centered” rather than paternalistic (Ishikawa et al. 2013, p. 150); (Epstein et al. 2005).

To help healthcare professionals with the challenge of “being responsive”, Agarwal and Murinson (2012) suggest that it can be helpful to distinguish patient archetypes and to develop ideas on how to respond to each of them efficiently:

In this new medical paradigm, the physician is often not the sole repository of medical information, which means that the patient–physician interaction is negotiated anew each time a knowledgeable patient is encountered, an unacceptably inefficient approach. […] A necessary future step in the further development of our new paradigm of patient–physician interaction includes a careful study of patient populations […]. By surveying patient populations with respect to autonomy, values, and medical knowledge, it will be possible to identify which patient types are most often seen. This will allow physicians to recognize patient types more quickly and understand more clearly which clinical approaches are most needed (Agarwal and Murinson 2012, p. 9–10).

Being responsive to a patient’s information needs also means that healthcare professionals carefully think about *how* they provide health information in any given encounters. When healthcare professionals are, for instance, faced with patients with literacy problems, they could use visual aids and analogies, and they need to be considerate in the language they use (Taylor 2009). Moreover, in situations in which patients are reluctant to be the sole decision-maker and therefore invoke the help of their doctor, healthcare professionals need not only provide patients with information, but should also provide patients with their interpretation of information (Sinding et al. 2010).

## Discussion

Three discourses of patient-centeredness have been presented on the basis of an analysis of a sample of scientific articles that discuss the meaning of patient-centeredness in the context of medical encounters. Like earlier research, the present article is rooted in an anti-essentialist approach to patient-centeredness and focuses on the ways in which the concept of patient-centeredness is brought into being through texts and practices (Hughes et al. 2008). Unlike earlier research, however, the focus was on highlighting differences rather then similarities.

As is in line with how Deetz (1996) presented discourses of organization science, the discourses of patient-centeredness need not be seen as ‘mutually exclusive’. Relational processes, such as conversations and texts like these, often involve a coming together of different discourses, which can lead to re-constructions and enrichments of discourses, and to the emergence of new discourses. Discourses can, for instance, “steal each other’s insights” (Deetz 1996). Consequently, the presented discourses are assumed to be fluid and open to change. At the same time, each discourse articulates a different set of arguments and practices that seem to be promoted when actors use the language of patient-centeredness. Or to put it differently, each discourse can be seen as a possible orientation to patient-centered encounters, and as a possible way of constructing the patients and healthcare professionals that participate in them.

Deetz (1996) argues that an important way to evaluate the results of a Discourse Analysis is by looking at how discourses pose problems for one another and hereby invite us to reflect on “differences that matter”. Indeed, I think that the discourses raise important moral questions for each other (Table 2).

**Table 2:**
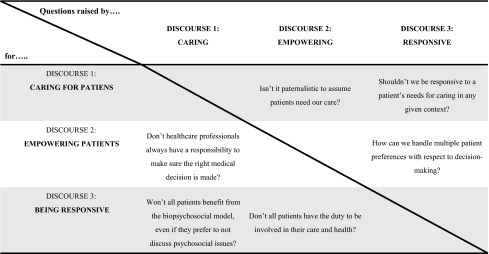
Questions raised by the discourses

One fundamental question that seems to underlie the questions in the table above is whether healthcare professionals should explore and then accept a patient’s wishes and abilities with respect to the content of medical encounters and his/her involvement in decision-making, or whether healthcare professionals should persuade patients into a certain direction. Let me say a bit more about how the discourses pose questions for each other and hereby help us to develop “critical insights” (Alvesson and Deetz 2000).

### Moral questions: conversations between the discourses

When patient-centeredness is constructed as a process of caring for patients, the focus tends to be on how the biopsychosocial model can be adopted (“discussing psychosocial issues, unless…”). After all, it is assumed that when the focus is purely on the patient’s disease, this could lead to wrong diagnoses, and treatments run the risk of being ineffective (see e.g. Mishler 1984; Bensing 2000). In addition, from a caring discourse it makes sense to argue that healthcare professionals should not refrain from their medical responsibilities. Salmon and Hall, for instance, argue that a discourse of empowerment mainly serves doctors (not patients), since it allows them “to withdraw from responsibilities for areas of patient need that are problematic for medicine” (Salmon and Hall 2003, p. 1969). Likewise, a recent article ‘subverts’ the popular assumption that the patient’s involvement in medical decision-making is particularly important in those cases in which there is great uncertainty surrounding the medical options and the “right” choice. In this text, the author argues that if patients are faced with complex considerations regarding unclear benefits and harms, they may benefit most from a recommendation from their healthcare professional (Fried 2016). Texts like these show how the arguments that are central in a discourse of caring raise relevant questions about patient-centeredness as empowering. It invites us to carefully consider the ‘when’ and ‘how’ of empowerment in relation to a patient’s need for good medical care.

When patient-centeredness is constructed as a process of “empowering patients”, the focus tends to be on how patients can be empowered – even if patients feel they lack the skills or do not seem up for it (“empowering patients, unless….”). Rather then taking the patient’s skills and values with respect to medical decision-making for granted, healthcare professionals are invited to explore how patients can be trained or coached (“empowered”) to make medical decisions and/or to manage their own health. See, for example, how Légaré and Witteman respond to arguments of vulnerable patients that cannot or should not be empowerment:

Although health care providers hold strong views regarding which patients want to, should, or even can engage in shared decision making, those views may be flawed. Surveys consistently indicate that patients want more engagement than they get. […] patients can learn communication skills and become increasingly confident in their ability to engage in decisions about their health. […] ethical and moral principles require that we not withhold it from vulnerable patients just because it may be more difficult to deliver it to them. Rather, we must find ways to deliver such care across the board (Légaré and Witteman 2013, p. 279).

Empowerment is often promoted because “it can lead to significant patient and clinician benefits, ultimately leading to better outcomes for individuals and societies” (Hannan and Webber 2007, p. 108). Moreover, patients that are involved in their care are expected to visit their doctor less frequently, which is often considered timely because of the increase of healthcare costs (Hughes et al. 2008). This argument illustrates how the discourses are linked to political interests and macro-level discourses, in this case empowerment is linked to a discourse of efficiency  and related to the interests of the healthcare system as a whole.

When patient-centeredness is discoursed as a challenge of responsive communication, ‘values’ such as empowerment and holistic caring are more or less presented as food for negotiation or for ‘shared reflection’. In other words, the focus is shifted to the medical encounter itself as an arena of negotiation. During their conversations, patients and healthcare professionals need to work out whether and how to discuss psychosocial issues and whether and how the patient is involved in medical decision-making (Illingworth 2010; Ishikawa et al. 2013). The local specifics of the encounter, including the skills and values of the participants at that particular moment, are thus centered over a more general ethical notion of ‘what is good’. The discourses of caring and empowerment raise questions about whether this isn’t a relativist approach, and suggest that patients could be persuaded to do what seems best for them (see e.g. Pulvirenti et al. 2014).

### Implications: the challenge of diversity

What to make of these different discourses? How to answer the questions they raise? The present relational constructionist philosophy suggests that we need to let go of the idea that there is a universal ground or “God’s eye view” from which to judge different discourses (Gergen 1999). Moreover, it suggests that discursive differences are both inescapable and can be appreciated. They are inescapable because conversations are likely to involve a coming together of multiple discourses. Different actors are likely to bring in different discourses that make sense in relation to the historical and cultural particularities of both the conversational context and their local “communities”. Discursive diversity needs to be appreciated, because it is vital to our well being (Falzon 1998). After all, a lack of diversity suggests a state of suppression of certain discourses over others, and such domination can stunt opportunities for the development of new insights and processes of innovation (Sampson 2008). Moreover, a lack of diversity often leads to taken-for-granted constructions of the “real and good”, which could mean that potentially interesting alternative constructions are being overlooked. Consequently, relational constructionism invites us to centre the challenge of working with differences in ways that are helpful to the local (groups of) actors involved. This requires that we accept differences, and that we start to critically examine the local realities we are making (or breaking) during conversations (Hosking 2007). This way we can develop “critical insights” into perhaps taken for granted discourses and their ethical and political consequences (Alvesson and Deetz 2000). Who is given certain rights, who are made responsible for different healthcare tasks, who are being silenced by a discourse? Do we feel this makes sense in this particular context, given our *local criteria* of ethics and the local interests of the actors involved? (Hosking and Pluut 2010). In sum, the present article suggests diversity and conflicts between discourses are both an inevitable and healthy aspect of social life. The challenge is to make conflict a “positive sum game”. The implications of this view are discussed below by using the example of projects that aim to make healthcare more patient-centered through the practice of “Health Information Exchange”.

### The case of Health Information Exchange

The greater availability of health information to patients has fuelled discussions on the role of patients in healthcare (Henwood et al. 2003). Consequently, it is not surprising that health information emerged out of the Discourse Analysis as one of the themes that is constructed differently in texts that construct patient-centeredness. Health Information Exchange (henceforth HIE) refers to the process of (electronically) sharing patient-level health data across a network of stakeholders (Vest and Gamm 2010; Shapiro and Kuperman 2011). Those who want to improve the availability of health information are faced with political challenges: who are given the rights and responsibilities to exchange which kind of information, for what reasons, and in what ways? As we have seen, the three discourses of patient-centeredness each provide a different answer to these questions. This explains why the ‘what, why, how, and for whom’ of HIE is often highly contested. Projects that aimed to facilitate HIE on a national scale, for instance, have suffered from unproductive, polarized debates (Garrety et al. 2014). One reason for this is that actors ignored the challenge of working with different discourses. To illustrate, those who evaluated a national HIE project in the Netherlands argued that a shared recognition of the patient’s interests did not bind actors together, but divided them (Twist et al. 2012). There seemed to be a false sense of consensus: all actors stated the HIE project needed to centre patients, but they meant different things by it. As Abma argued, there is a serious risk in using abstract concepts such as patient-centeredness:

When vague and abstract […] concepts […] have to be implemented, the consensus often appears to be a superficial one. Under the outer layer of homogeneity one finds a broad spectrum of meanings, which not infrequently lead to heated discussions and a confusion of tongues (Abma 2000, p. 199-200).

Discussions on whatever sort of patient-centered innovation run the risk of becoming unproductive if they fail to acknowledge that patient-centeredness can be constructed differently by different (groups of) actors, in relation to their varying norms, values, and interests in a local project. Consequently, rather than assuming that we all want what is best for patients, we need to anticipate dissensus and need to explore how we can deal with different discourses of patient-centeredness in relation to the local challenges we are facing.

As Gergen argues, “if dialogue is to proceed successfully, it is critical that the other understands who we are and what we stand for” (Gergen 1999, p. 158). This implies that when particular HIE solutions are being debated, it is vital that actors link different arguments to different discourses, and that they gain insights into how these discourses makes sense in relation to the interests, norms and values of the (groups of) actors that are involved. The discourses that are presented in this article hopefully sensitize practitioners in healthcare to different discourses of patient-centeredness and help them gain an understanding for why different actors promote particular (HIE) practices. Developing such critical insights is an important first step towards making conflicts on patient-centered HIE a “positive sum game” (Abma 2000; Alvesson and Deetz 2000). It means that the different stakeholders are starting to build productive working relations that are rooted in an understanding of the multiple local “rationalities” and a recognition of the communal challenge of working with diversity in fruitful ways.

### Limitations

I do not wish to claim that this article presents all possible or all popular constructions of patient-centeredness. Hopefully it does, however, paint an interesting and colourful picture of how scientific texts that discuss the meaning of patient-centered encounters construct this multi-faced concept differently. Some may feel that additional themes or arguments should have been included in the overview of discourses. Likewise, some perhaps argue that interesting articles on the meaning of patient-centeredness have been overlooked and should have been included in the analysis. In that case, I would invite readers to join and enrich the dialogue on how we can construct patient-centeredness. I prefer to not see this article as the end of an analytical process, but as a potentially useful starting point for dialogues on how to deal with moral dilemmas and conflicts over patient-centered care practices.

### Future research

Future research could broaden the scope of the present discourse analysis to include other kinds of “centeredness”, such as client-, family- person- and relationship-centeredness (Hughes et al. 2008). Perhaps a focus on different constructions of these concepts highlights other relevant dilemmas and suggests additional themes that can be opened up for reflection. Likewise, future research could explore how actors other than scientists (policy makers, doctors, patients, managers) construct patient-centeredness in different contexts and can hereby bring additional candidates for reflection to the fore.

Second, future research could focus on how the results of the presented analysis can be used in ways that stimulate reflexivity and that help actors to make conflicts over patient-centered practices productive. It could, for instance, explore how reflexive workshops can help practitioners (e.g. healthcare professionals, patients, managers, policymakers, researchers) to reflect on different discourses and the challenge of dissensus, hereby developing “practical theories” as to how to deal with diversity within local working contexts (Cunliffe 2003; Abma 2000).

## Conclusions

The discourse analysis shows how patient-centeredness is a moral concept (Duggan et al. 2006) and it sensitizes us to important ethical questions. Hence, I would argue that generic models of patient-centeredness (e.g. Mead and Bower 2000) mask multiplicity and fail to address the normative differences in ideas about how doctor and patient should carry out their relationship. When we approach patient-centeredness as a relationally constructed concept and accept there is no “God’s eye view” from which to judge different discourses, this invites us to shift our attention to the challenge of dealing with different constructions of patient-centered care in local contexts.

Diversity and conflicts are inevitable and healthy aspects of social life. Repressing conflicts can lead to boredom, the storage of anger, and the negation of attainable goals. If we are able to keep conflicts healthy, on the other hand, they may spark creativity and can open the way for innovation (Abma 2000). Let us therefore accept different constructions of patient-centeredness, and focus on the challenge of handling diversity. In this context, we can embrace reflexivity as a conversational recourse. We can communally reflect upon discursive differences in relation to the norms, values, and interests of the different local (groups of) actors that are involved (Hosking and Pluut 2010; Johnson and Duberley 2003). This way, practitioners in healthcare can become more empowered to articulate and develop their discourses of patient-centeredness, and to facilitate processes that aim to improve the patient-centeredness of local healthcare contexts in ways that make sense in relation to local criteria of healthcare quality and ethics.

## Appendix

Below is a list of the 34 scientific journal articles that were included in the discourse analysis of texts that discuss the meaning of patient-centeredness.

1. Abley, C. (2012). Responding to vulnerability in old age: Patient-centred care. *Nursing Standard*, *27*(9), 42–46.
2. Agarwal, A. K., & Murinson, B. B. (2012). New dimensions in patient-physician interaction: Values, autonomy, and medical information in the patient-centered clinical encounter. *Rambam Maimonides Medical Journal*, *3*(3), e0017.
3. Anderson, R. M., & Funnell, M. M. (2010). Patient empowerment: Myths and misconceptions. *Patient Education and Counseling*, *79*(3), 277–282.
4. Aujoulat, I., d’Hoore, W., & Deccache, A. (2007). Patient empowerment in theory and practice: Polysemy or cacophony? *Patient Education and Counseling*, *66*(1), 13–20.
5. Badcott, D. (2005). The expert patient: Valid recognition or false hope? *Medicine, Health Care, and Philosophy*, *8*(2), 173–178.
6. Barry, M. J., & Edgman-Levitan, S. (2012). Shared decision making: The pinnacle of patient-centered care. *New England Journal of Medicine*, *366*(9), 780–781.
7. Bensing, J. (2000). Bridging the gap: The separate worlds of evidence-based medicine and patient-centered medicine. *Patient Education and Counseling*, *39*(1), 17–25.
8. Bergman, A. A., & Connaughton, S. L. (2013). What is patient-centered care really? Voices of hispanic prenatal patients. *Health Communication*, *28*(8), 789-799.
9. Berwick, D. M. (2009). What ‘patient-centered’ should mean: Confessions of an extremist. *Health Affairs (Project Hope)*, *28*(4), w555–w565.
10. Davis, K., Schoenbaum, S. C., & Audet, A. M. (2005). A 2020 vision of patient-centered primary care. *Journal of General Internal Medicine*, *20*(10), 953–957.
11. De Haes, H. (2006). Dilemmas in patient centeredness and shared decision making: A case for vulnerability. *Patient Education and Counseling, 62*(3), 291–298.
12. Epstein, R. M., Franks, P., Fiscella, K., Shields, C. G., Meldrum, S. C., Kravitz, R. L., & Duberstein, P. R. (2005). Measuring patient-centered communication in patient-physician consultations: Theoretical and practical issues. *Social Science & Medicine*, *61*(7), 1516–1528.
13. Fineberg, H. V. (2012). From shared decision making to patient-centered decision making. *Israel Journal of Health Policy Research*, *1*(1), 1–2.
14. Henwood, F., Wyatt, S., Hart, A., & Smith, J. (2003). ‘Ignorance is bliss sometimes’: Constraints on the emergence of the ‘informed patient’ in the changing landscapes of health information. *Sociology of Health & Illness, 25*(6), 589–607.
15. Hobbs, J. L. (2009). A dimensional analysis of patient-centered care. Nursing Research, 58(1), 52–62.
16. Holmström, I., & Röing, M. (2010). The relation between patient-centeredness and patient empowerment: A discussion on concepts. *Patient Education and Counseling, 79*(2), 167–172.
17. Hudon, C., Fortin, M., Haggerty, J., Loignon, C., Lambert, M., & Poitras, M. E. (2012). Patient-centered care in chronic disease management: A thematic analysis of the literature in family medicine. *Patient Education and Counseling*, *88*(2), 170–6.
18. Illingworth, R. (2010). What does patient-centred mean in relation to the consultation? *The Clinical Teacher*, *7*(2), 116–120.
19. Ishikawa, H., Hashimoto, H., & Kiuchi, T. (2013). The evolving concept of “patient-centeredness” in patient-physician communication research. *Social Science & Medicine*, *96*, 147–53.
20. Jayadevappa, R., & Chhatre, S. (2011). Patient centered care-a conceptual model and review of the state of the art. *The Open Health Services and Policy Journal*, *4*(1), 15–25.
21. Lawrence, M., & Kinn, S. (2012). Defining and measuring patient-centred care: An example from a mixed-methods systematic review of the stroke literature. *Health Expectations*, *15*(3), 295–326.
22. Légaré, F., & Witteman, H. O. (2013). Shared decision making: Examining key elements and barriers to adoption into routine clinical practice. *Health Affairs*, *32*(2), 276–284.
23. Lusk, J. M., & Fater, K. (2013). A concept analysis of patient-centered care. *Nursing Forum*, *48*(2), 89–98.
24. Makoul, G., & Clayman, M. L. (2006). An integrative model of shared decision making in medical encounters. *Patient Education and Counseling*, *60*(3), 301–312.
25. Moreau, A., Carol, L., Dedianne, M. C., Dupraz, C., Perdrix, C., Lainé, X., & Souweine, G. (2012). What perceptions do patients have of decision making (DM)? Toward an integrative patient-centered care model. A qualitative study using focus-group interviews. *Patient Education and Counseling*, *87*(2), 206–211.
26. Morgan, S., & Yoder, L. H. (2012). A concept analysis of person-centered care. *Journal of Holistic Nursing: Official Journal of the American Holistic Nurses’ Association*, *30*(1), 6–15.
27. Moumjid, N., Gafni, A., Brémond, A., & Carrère, M. O. (2007). Shared decision making in the medical encounter: Are we all talking about the same thing? *Medical Decision Making: An International Journal of the Society for Medical Decision Making*, *27*(5), 539–546.
28. Piper, S. (2010). Patient empowerment: Emancipatory or technological practice? *Patient Education and Counseling*, *79*(2), 173–177.
29. Pulvirenti, M., McMillan, J., & Lawn, S. (2014). Empowerment, patient centred care and self-management. *Health Expectations*, *17*(3), 303–310.
30. Robinson, J. H., Callister, L. C., Berry, J. A., & Dearing, K. A. (2008). Patient-centered care and adherence: Definitions and applications to improve outcomes. *Journal of the American Academy of Nurse Practitioners*, *20*(12), 600–607.
31. Sacristán, J. A. (2013). Patient-centered medicine and patient-oriented research: Improving health outcomes for individual patients. *BMC Medical Informatics and Decision Making*, *13*, 1–8.
32. Sinding, C., Hudak, P., Wiernikowski, J., Aronson, J., Miller, P., Gould, J., & Fitzpatrick-Lewis, D. (2010). “I like to be an informed person but…” Negotiating responsibility for treatment decisions in cancer care. *Social Science & Medicine*, *71*(6), 1094–1101.
33. Tanenbaum, S. J. (2013). What is patient-centered care? A typology of models and missions. *Health Care Analysis.*
34. Taylor, K. (2009). Paternalism, participation and partnership - the evolution of patient centeredness in the consultation. *Patient Education and Counseling*, *74*(2), 150–155.
